# Comparison of Longitudinal and Transverse Approaches for Ultrasound-Guided Lumbar Erector Spinae Plane Block in Feline Cadavers

**DOI:** 10.3390/vetsci13060569

**Published:** 2026-06-10

**Authors:** Sara Carrillo-Flores, Marta Soler, Francisco Gil, Gonzalo Polo-Paredes, Francisco G. Laredo, Amalia Agut, Eliseo Belda

**Affiliations:** 1 Hospital Veterinario Universidad de Murcia, 30100 Murcia, Spain; sacarriflores@gmail.com (S.C.-F.); mtasoler@um.es (M.S.); gpolo@um.es (G.P.-P.); laredo@um.es (F.G.L.); amalia@um.es (A.A.); 2 Departamento de Medicina y Cirugía Animal, Facultad de Veterinaria, Universidad de Murcia, 30100 Murcia, Spain; 3 Departamento de Anatomía y Anatomía Patológica Comparada, Facultad de Veterinaria, Universidad de Murcia, 30100 Murcia, Spain; cano@um.es

**Keywords:** erector spinae, ultrasound-guided anesthesia, locoregional anesthesia, dorsal branches of the spinal nerves, third lumbar vertebra (L3), ventral branches of the spinal nerves

## Abstract

The ultrasound-guided erector spinae plane (ESP) block is a locoregional anesthesia technique primarily aimed at providing analgesia to structures innervated by the dorsal branches of the spinal nerves (DBSN). While this block has been widely studied in dogs, evidence in cats is limited, and only a few cadaveric studies have addressed the lumbar region. The aim of this study was to compare the injectate distribution and staining of the DBSN following ultrasound-guided lumbar ESP blocks performed using either a longitudinal or transverse approach in feline cadavers. A total of 15 feline cadavers were included, with 3 used for anatomical dissection and 12 for ultrasound-guided injections (24 sides). Injections were performed at the level of the third lumbar vertebra (L3) using a mixture of methylene blue, lidocaine, and iopromide (0.4 mL kg^−1^ per side). Needle placement and injectate spread were guided and confirmed by ultrasonography, followed by computed tomography (CT) and anatomical dissection to evaluate contrast and dye distribution. CT images revealed longitudinal spread of the contrast in all injections, with the transverse approach producing exclusively longitudinal distribution and the longitudinal approach showing occasional ventral spread beneath the transverse processes (25% of sides). Dissections demonstrated staining of a median of 2 DBSN per side for both approaches, predominantly L2–L3, with rare partial staining of ventral branches (VBSN) observed only with the longitudinal approach. The sympathetic trunk was not stained in any injection. No statistically significant differences were observed between approaches, except for L1 DBSN staining, which was identified significantly more frequently with the transverse approach (*p* = 0.033). These findings indicate that ultrasound-guided lumbar ESP block is anatomically feasible in feline cadavers and that both longitudinal and transverse approaches can result in injectate distribution to the DBSN.

## Simple Summary

The erector spinae (ESP) complex is part of the epaxial musculature and is enclosed by the dorsal layer of the thoracolumbar fascia. The dorsal branches of the spinal nerves (DBSN) provide innervation to the epaxial musculature, spinous processes, vertebral laminae, and the skin of the dorsum. The ultrasound-guided ESP block is a locoregional anesthesia technique intended to desensitize the DBSN to provide analgesia for chronic back pain and spinal surgery. However, in human medicine its use has also been described for thoracic and abdominal surgical procedures. A previous study in canine cadavers suggested that the transverse ultrasound-guided lumbar approach to the ESP block may be more suitable than the longitudinal approach. This comparison has not been conducted in feline cadavers. The aim of this cadaveric study was to compare the transverse and longitudinal ultrasound-guided lumbar ESP block in the feline species. Fifteen cadavers were included: three were used to examine the relevant anatomical structures, and twelve for the ultrasound-guided approaches. Either the transverse or longitudinal ESP block was randomly performed on each side of the cadavers. A mixture of methylene blue, lidocaine and iopromide contrast medium (0.4 mL kg^−1^) per side was injected at the level of the third lumbar vertebra (L3). Following injection, the cadavers underwent computed tomography and anatomical dissection. The results showed staining of the epaxial musculature and DBSN, with no differences between approaches. Anecdotally, the ventral branch of L3 was stained on two sides using the longitudinal approach. These findings indicate that both the longitudinal and transverse approaches to the ESP block are feasible and produce similar outcomes in feline cadavers.

## 1. Introduction

The epaxial musculature of the spine includes longitudinal muscle groups located dorsal to the vertebral column and ribs. These muscles act as extensors, stabilizers, and contribute to lateral movements of the trunk. One of the most relevant muscular systems within the epaxial musculature is the erector spinae, which comprises the iliocostal, longissimus, and spinal muscles. All of them are innervated by the dorsal branches of the spinal nerves (DBSN) [1].

The erector spinae plane (ESP) block is an interfascial locoregional anesthesia technique traditionally performed in the fascial plane between the vertebral transverse processes and the erector spinae muscles [2,3,4]. It was first described in humans by Forero et al. (2016), who reported a longitudinal approach at the thoracic level using a linear transducer [5]. In humans, this block has also been performed at the lumbar level using different approaches, including longitudinal and transverse techniques, to improve technical feasibility and procedural safety [6,7,8,9,10,11]. The ESP block was initially described as a technique to provide analgesia to structures innervated by the DBSN [12,13]. However, it is now also commonly used to control nociception in hypaxial regions innervated by the ventral branches of the spinal nerves (VBSN) [14,15,16]. Nonetheless, the mechanism of action of the ESP block at this level is still not fully understood, as cadaveric studies have produced inconsistent evidence of anesthetic spread to the VBSN [17,18].

In veterinary medicine, this block has been described in several species, including horses, rabbits, cows, pigs, dogs and cats [3,19,20,21,22,23]. As previously described in humans, the mechanism of action of this block remains controversial. Some cadaveric studies have demonstrated spread limited to the DBSN [3,24], whereas others have reported involvement of both the DBSN and the VBSN [23,25,26]. In canine cadaveric studies, both the longitudinal and transverse approaches have been evaluated [3,4,25]. These studies concluded that the transverse approach showed better injectate distribution at the lumbar and thoracolumbar level, but not in the thoracic area. In the feline species, only the longitudinal approach has been described in two cadaveric studies, one at the lumbar and one at the thoracic level [23,26].

The clinical use of this block, in veterinary anesthesia, is primarily aimed at providing analgesia for surgical procedures involving structures innervated by the DBSN, such as hemilaminectomy in dogs [27,28,29,30,31,32,33,34], spinal decompression surgery in rabbits [22], lumbar spinous process osteotomy in horses [35], and spinal surgery in cats [36]. However, two case reports have described its use in procedures involving the VBSN, specifically sternotomy and pancreatitis in dogs [37,38].

The primary aim of this study was to investigate the anatomy of the lumbar epaxial region and to compare the spread of the injectate and the staining of the DBSN following administration of a mixture of methylene blue (MB) and iopromide via either a longitudinal or transverse approach to the ESP block at the level of the third lumbar vertebra (L3) in feline cadavers. A secondary objective was to determine whether the injectate extended beneath the middle layer of the thoracolumbar fascia and whether the VBSN were stained. We hypothesized that the transverse approach would result in a more extensive distribution of the injectate and that a proportion of the VBSN would be stained.

## 2. Materials and Methods

This research was approved by the University of Murcia’s Biosecurity Committee in Experimentation (CBE 692/2025).

### 2.1. Animals

A total of fifteen feline cadavers were used (Table 1). The cadavers were donated to the University of Murcia through the Donation Program of the Veterinary Faculty. The cause of death was not related to the study. After donation, the cadavers were frozen and subsequently thawed for 48 h at room temperature. Following thawing, radiographic examinations of the lumbar region were performed to verify the absence of congenital anomalies or acquired alterations that could affect the study.

Three of the fifteen cat cadavers were used for a preliminary anatomical study, while the remaining twelve were employed to compare the longitudinal and transverse approaches.

**Table 1:** Cat cadaver’s demographics and ESP block approaches used for each side.

Cat	Sex	Weight (kg)	BCS ^1^	ESP Block	
				Side	Approach
Cat 1	Neutered Male	3.22	2/5	Right	Longitudinal
				Left	Transverse
Cat 2	Female	2.30	2/5	Right	Transverse
				Left	Longitudinal
Cat 3	Neutered male	4.45	3/5	Right	Transverse
				Left	Longitudinal
Cat 4	Intact male	1.70	2/5	Right	Transverse
				Left	Longitudinal
Cat 5	Neutered male	2.70	2/5	Right	Longitudinal
				Left	Transverse
Cat 6	Neutered male	2.90	3/5	Right	Longitudinal
				Left	Transverse
Cat 7	Intact male	4.47	3/5	Right	Longitudinal
				Left	Transverse
Cat 8	Neutered male	3.60	3/5	Right	Transverse
				Left	Longitudinal
Cat 9	Intact male	7.00	4/5	Right	Longitudinal
				Left	Transverse
Cat 10	Female	4.70	4/5	Right	Transverse
				Left	Longitudinal
Cat 11	Female	2.50	3/5	Right	Longitudinal
				Left	Transverse
Cat 12	Intact male	4.00	3/5	Right	Transverse
				Left	Longitudinal

^1^ BCS: Body condition score.

### 2.2. Anatomical Study

**Figure 1:**
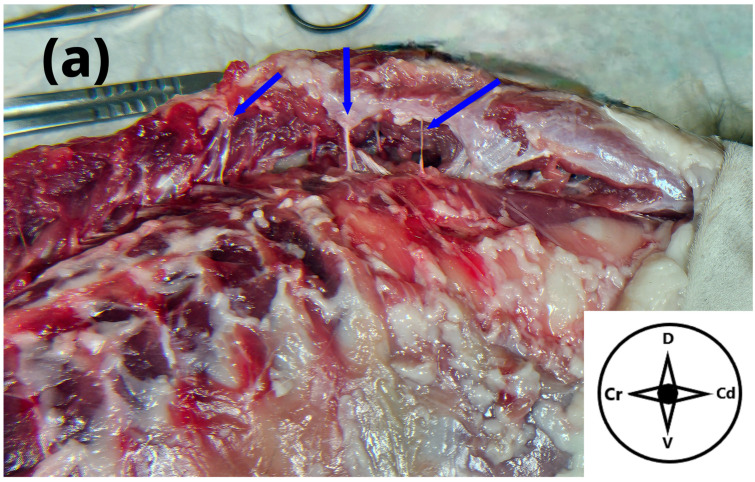

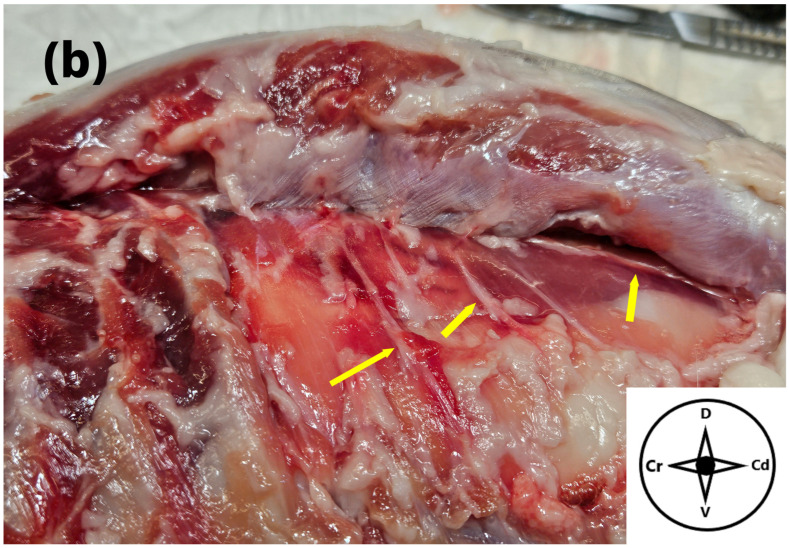
Anatomical dissection of the lumbar epaxial and paravertebral region in a feline cadaver. (**a**) Dorsal view of the dissected lumbar region showing the epaxial musculature and the dorsal branches of the spinal nerves from the first (L1) to the third (L3) lumbar vertebrae (blue arrows). (**b**) Ventral view following abdominal cavity access and viscera retraction, allowing identification of the ventral branches of the spinal nerves from L1 to L3 (yellow arrows) within the paravertebral musculature. Cr, cranial; Cd, caudal; D, dorsal; V, ventral.

For the anatomical dissection, the cadavers were placed in the lateral recumbency, and a longitudinal incision was made dorsal to the transverse processes, extending from the sixth lumbar vertebra to the eleventh thoracic vertebra (T11). After dissecting the skin down to the linea alba, the latissimus dorsi and the external oblique muscles were observed. Once both muscles were removed, the thoracolumbar fascia was dissected, allowing visualization of the iliocostalis, longissimus, and spinalis–semispinalis muscles. To access the DBSN, the longissimus muscle was dissected. The same procedure was performed on both sides (Figure 1).

To visualize the VBSN and sympathetic trunk, the cadavers were placed in dorsal recumbency, and a longitudinal incision was made along the linea alba. After entering the abdominal cavity, the abdominal viscera were retracted, allowing identification of the VBSN on both sides of the vertebral column within the abdominal musculature. The sympathetic trunk was also visualized in the most dorsal region, on the left side adjacent to the aorta, and on the right side adjacent to the caudal vena cava.

### 2.3. Ultrasound-Guided Technique

Twelve cat cadavers were used to perform bilateral ESP approaches, resulting in a total of 24 injections. Each cadaver received both approaches. The side where the longitudinal or transversal approach were performed was randomly assigned (https://www.random.org, accessed on 24 April 2025).

Injections were performed at the level of L3 using a linear ultrasound probe (3–13 Hz, SL1543, MyLabGamma Vet, Esaote, Florence, Italy). A mixture of 0.4 mL kg^−1^ per side of MB (Panreac Química, Appli-138 Chem, Castellar del Vallès, Spain), diluted with lidocaine (lidocaine 2%, B. Braun Medical S.A., Rubí, Spain) and iopromide (300 mgI mL^−1^, Ultravist 300, Bayer, Berlin, Germany) was used, achieving a final MB concentration of 0.5% in a 50:50 lidocaine/iopromide mixture.

**Figure 2:**
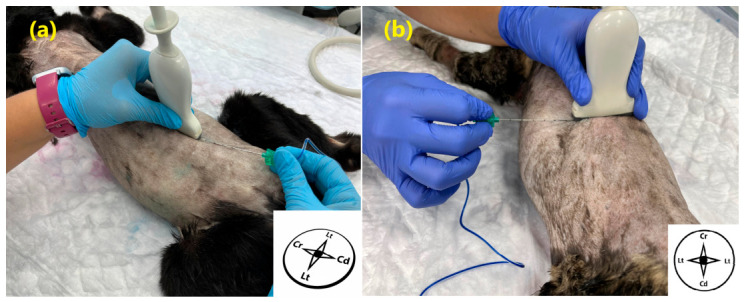
(**a**) Longitudinal approach, with the transducer placed dorsally over the spine in a parasagittal plane and the needle directed in a caudocranial orientation. (**b**) Transverse approach, with the transducer positioned perpendicular to the spine and the needle oriented in a lateromedial direction. Cr, cranial; Cd, caudal; Lt, lateral.

To perform the approaches, all cadavers were positioned in sternal recumbency (Figure 2). In the longitudinal approach, the last rib was first identified ultrasonographically, and the transducer was moved dorsally to visualize the last thoracic vertebra (T13). From this point, the vertebrae were counted caudally to the level of L3. The transverse process of L3 was identified as a hyperechoic slightly convex line with acoustic shadowing. An echogenic needle (Ultraplex^®^ 20 G, 50 mm, 30°, B. Braun, Melsungen, Germany) was inserted in-plane in a caudocranial direction, advancing through the epaxial musculature until the needle tip contacted the transverse process of L3. To confirm correct needle placement, a test volume of the prepared solution was injected (0.1 mL). When the ultrasonographic image was consistent with the expected distribution (anechoic spread between the lumbosacral common muscle group and the transverse process of L3), the remainder of the injectate was administered.

**Figure 3:**
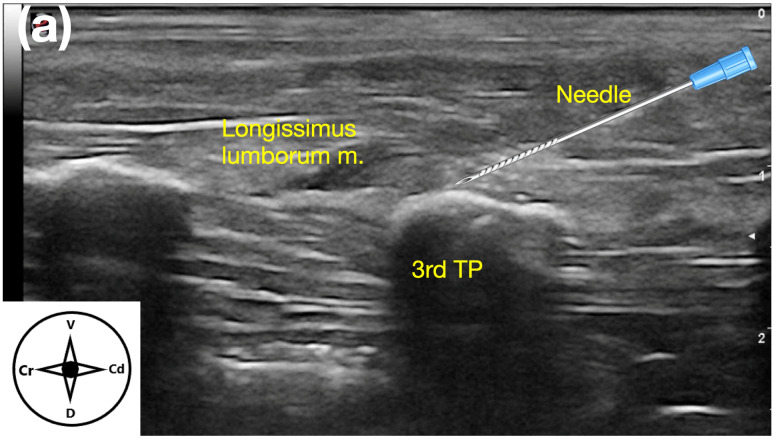

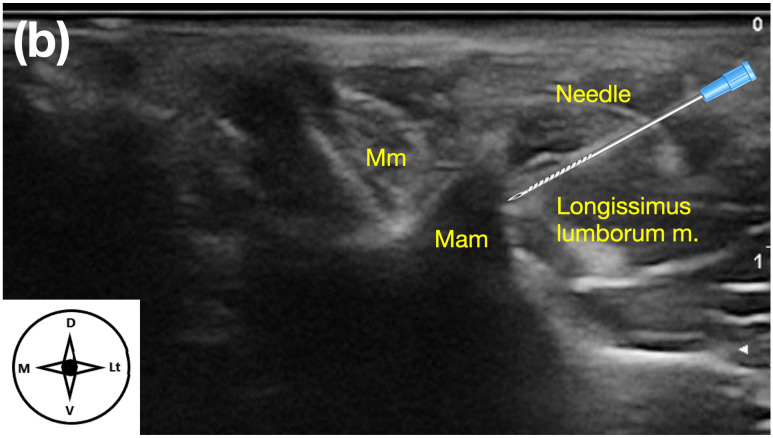
(**a**) Ultrasound image of the longitudinal approach, showing the epaxial musculature and reference landmarks with the transducer oriented in a longitudinal plane. The needle trajectory is directed in a caudocranial direction. (**b**) Ultrasound image of the transverse approach, illustrating the epaxial musculature and relevant landmarks with the transducer oriented perpendicular to the spine. TP, transverse process; Mam, mamillary process; Mm, multifidus muscle; Cr, cranial; Cd, caudal; D, dorsal; V, ventral; M, medial; Lt, lateral.

For the transverse approach, L3 was identified ultrasonographically using the same procedure as in the longitudinal approach. The transducer was then rotated anticlockwise into a transverse orientation to allow visualization of the mammillary process of L3. Once identified, the needle was inserted in-plane in a lateromedial direction until the needle tip contacted the lateral border of the mammillary process. To verify correct positioning of the needle tip, a small amount of the total volume was injected (0.1 mL). In cases where the distribution was consistent with the expected pattern (anechoic spread between the mammillary process and the lumbosacral common muscle group), the remaining injectate was delivered (Figure 3).

In both approaches, needle visualization was considered adequate when the entire needle was visualized “in-plane” throughout the procedure. If any portion of the needle could not be identified on the ultrasound image, needle visualization was considered inadequate.

### 2.4. Computed Tomography (CT) Study

Fifteen to twenty minutes after the injections, a helical CT examination was conducted from the T11 to the seventh lumbar vertebra (L7) using a 16-slice multidetector CT scanner (Revolution, General Electric Healthcare, Madrid, Spain). The cadavers were positioned in dorsal recumbency with the forelimbs and hindlimbs extended cranial and caudal respectively. Scanning parameters included a collimator pitch of 1 and a slice thickness of 2.5 mm. Images were reconstructed with a 1.25 mm overlapping interval. Both tube voltage and current were set at 120 kVp and 120 mA, respectively. Standard bone and soft tissue reconstruction algorithms were applied.

The reconstructed images were independently evaluated by two imaging specialists (M.S. and A.A.) to assess the distribution of the contrast medium; the evaluators were not blinded to the experimental conditions.

### 2.5. Spread Study

After performing the CT scan, an anatomical study of the structures stained with MB was conducted. The anatomical dissection was carried out as previously described. The nerves were classified as stained, partially stained, or unstained according to the extent of staining: stained when ≥1 cm of their whole circumference was dyed, partially stained when the staining involved <1 cm or not the whole circumference, and unstained in the absence of coloration. The number of vertebral bodies along which the sympathetic trunk was stained was recorded. The same evaluation criteria were applied to the sympathetic trunk. All dissections were performed by the same operators (S.C.-F. and F.G.), who were not blinded to the experimental conditions.

### 2.6. Statistical Analysis

Statistical analysis was performed using IBM SPSS Statistics for Windows, version 21.0 (IBM Corp., Armonk, NY, USA). Data normality was assessed using the Shapiro–Wilk test and included contrast medium distribution, the percentage of vertebral bodies reached by the contrast medium, the number of stained DBSN and VBSN, and the percentage of staining for each DBSN and VBSN. Non-normally distributed variables are presented as median and range for contrast medium distribution and the number of stained DBSN and VBSN, whereas categorical data are expressed as percentages, including the vertebral bodies reached by the contrast medium and the proportion of stained DBSN and VBSN. Comparisons between the longitudinal and transverse approaches were performed using the Mann–Whitney U test. No corrections for multiple comparisons were applied. Statistical significance was set at *p* < 0.05.

## 3. Results

### 3.1. Anatomical Study

Three feline cadavers were used for this phase: two males and one female, with a median body weight of 3.8 (1.98–4.38) kg. After skin removal, the latissimus dorsi was identified in the dorsal region, while the external abdominal oblique was observed more laterally. Both muscles were reflected to expose the dorsal layer of the thoracolumbar fascia. This layer was then incised to reveal the erector spinae muscle group. To visualize the DBSN, a dissection was performed between the longissimus and iliocostalis muscles. At the level of T13 the longissimus and iliocostalis muscles joined and, together with the spinalis–semispinalis muscles, constituted the common lumbosacral muscle group. No distinction between lateral and medial branches of the DBSN was identified.

To visualize the VBSN and the sympathetic trunk, the abdominal cavity was accessed through a longitudinal incision of the midline of the ventral abdominal wall. Following skin and subcutaneous tissue incision, the linea alba was opened to enter the peritoneal cavity. The abdominal viscera were retracted to expose the retroperitoneal space. The sympathetic trunk was visualized dorsally, positioned adjacent to the aorta on the left side and alongside the caudal vena cava on the right side. Then, the quadratus lumborum, psoas major, and psoas minor muscles were identified as anatomical landmarks. The VBSN were located emerging from the psoas major, with the quadratus lumborum positioned lateral and slightly dorsal, and the psoas minor lying medial to the psoas major. This approach allowed precise identification of the VBSN while preserving the surrounding musculature and fascia.

### 3.2. Ultrasound-Guided Technique

A total of twenty-four injections were performed using twelve cat cadavers (one injection per side), evenly divided between the longitudinal and transverse groups. All injections were performed at the level of L3. The median body weight of the animals used was 3.11 (1.6–6.98).

Needle visualization was considered adequate in all injections (24/24) in both groups. The mixture of MB and contrast medium was visualized ultrasonographically as an anechoic fluid pocket separating the epaxial musculature from the transverse process or the mammillary process at the injection site. Partial intramuscular distribution of the injected material was observed in all injections in both groups.

### 3.3. Computed Tomography (CT) Study

**Figure 4:**
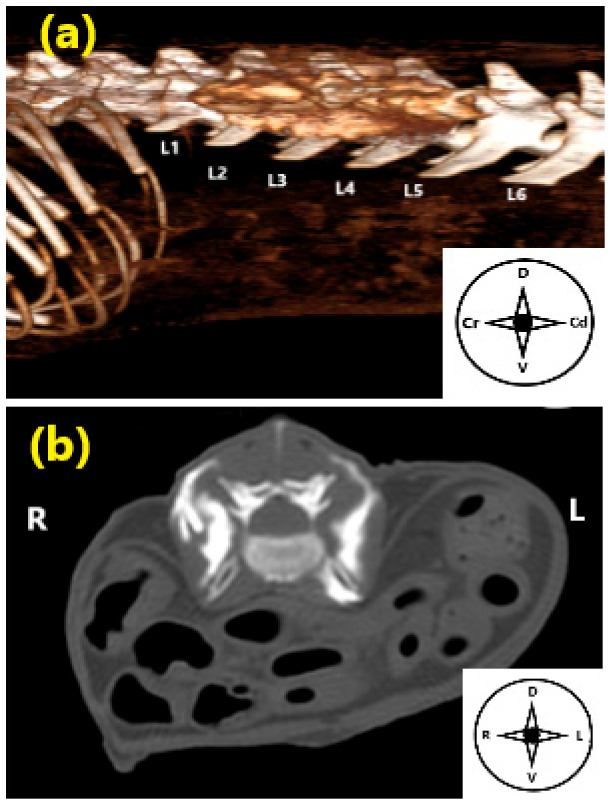
Computed tomography (CT) images illustrating the distribution of contrast medium following erector spinae plane injection at the level of the third lumbar vertebra (L3) in a feline cadaver. (**a**) Three-dimensional CT reconstruction in lateral view showing the spread of contrast medium within the lumbar epaxial musculature. (**b**) Transverse CT image at the same level demonstrating contrast distribution within the epaxial musculature and extending ventrally beneath the lumbar vertebra. Cr, cranial; Cd, caudal; D, dorsal; V, ventral; L, left; R, right.

Contrast medium was observed in the target region (L3) in 100% (24/24) of the injections. The distribution of the contrast medium was longitudinal in all injections performed using the transverse approach (12/12). The longitudinal approach showed a longitudinal spread in all the sides (12/12). However, in this approach, contrast medium was also ventrally distributed (under the transverse processes of L3) in 25% (3/12) of sides (Figure 4).

The spread of contrast medium had a median of 4 (3–5) and 4 (3–5) vertebral bodies in the longitudinal and the transverse approach respectively, spanning from the first lumbar vertebra (L1) to the fifth lumbar vertebra (L5) in the longitudinal approach and from T13 to L5 in the transverse approach (Table 2).

In the longitudinal approach, the vertebral bodies showing the greatest presence of contrast medium were the second (L2), L3, and fourth (L4) lumbar vertebrae, with contrast medium observed in (12/12) of the sides. The L5 showed contrast medium in (6/12) of the sides, whereas L1 had the lowest presence of contrast medium, detected in (4/12) of the sides.

**Figure 5:**
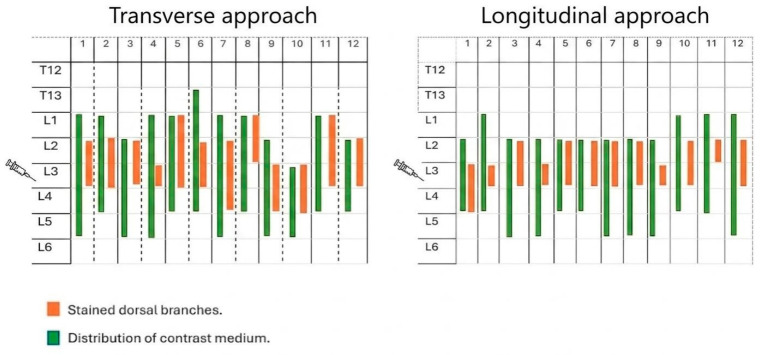
Number of vertebral bodies (CT distribution of contrast medium) and stained dorsal branches of the spinal nerves in twelve feline cadavers following injection of a mixture of 0.4 mL kg^−1^ methylene blue, lidocaine, and iopromide.

For the transverse approach, contrast medium was observed at L3 and L4 in (12/12) of the sides, followed by L2 in (11/12), L1 in (8/12), and L5 in (6/12). The T13 showed the lowest presence of contrast, observed in (1/12) of the sides (Table 3). No statistically significant differences were found between both approaches (Figure 5). Contrast medium was not detected in the epidural space in any of the cadaver.

### 3.4. Spread Study

Methylene blue was observed in the epaxial musculature in all injected sides (24/24). A median of 2 (1–2) and 2 (1–3) DBSN were stained in the longitudinal and the transverse approaches respectively (Table 2). The stained DBSN in the longitudinal approach were L2 in (8/12) of the sides, L3 in (11/12), and L4, which was stained in (1/12) and partially stained in (1/12). In the transverse approach, the stained DBSN were L1 in (3/12), with partial staining observed in (1/12) of the sides, L2 in (9/12), L3 in (11/12), and L4 in (3/12) (Table 4). Statistically significant differences between approaches were observed only for L1 (*p* = 0.033) (Figure 5).

**Figure 6:**
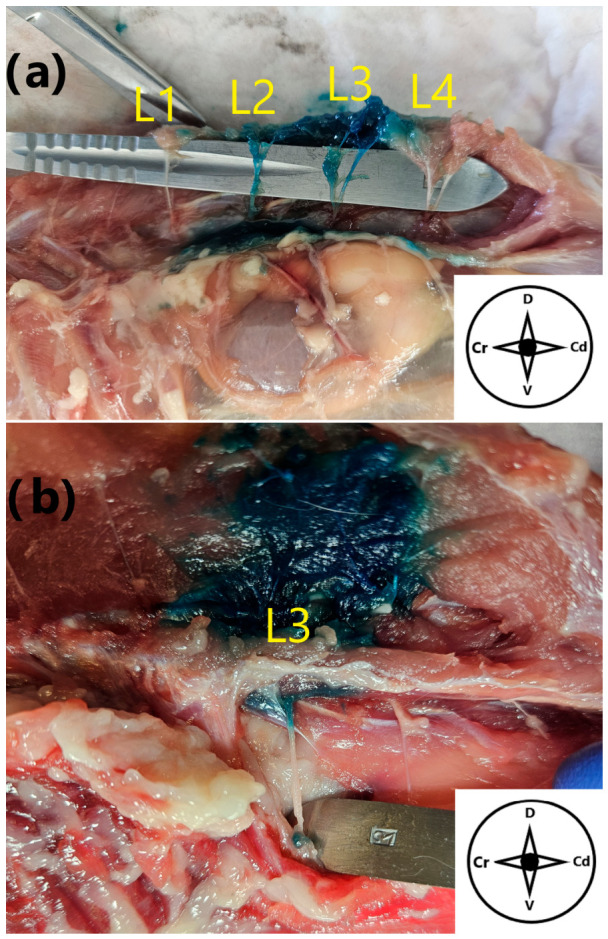
Anatomical dissections illustrating the distribution of methylene blue staining following the lumbar erector spinae plane block in a feline cadaver. (**a**) Dorsal dissection of the lumbar epaxial musculature of the dorsal branches of the spinal nerves from the first (L1) to the fourth lumbar (L4) vertebrae. (**b**) Ventral dissection of the lumbar paravertebral region showing the ventral branches at the level of the second (L2) and third lumbar (L3) vertebrae. Cr, cranial; Cd, caudal; D, dorsal; V, ventral.

Regarding the VBSN, staining was observed only in the longitudinal approach, with a median of 0 (0–2) (Table 2). The affected branches were L1, L2, and L3, all of which showed partial staining in 8.3% (1/12) of the sides. The sympathetic trunk was not stained in any of the injections (Figure 6).

## 4. Discussion

According to our results, the hypothesis that the transverse approach would result in superior spread of MB and iopromide must be rejected. A similar staining pattern and number of DBSN were observed following the injection of 0.4 mL kg^−1^ of injectate at the level of L3, using both longitudinal and transverse approaches. On the contrary, Medina-Serra et al. (2021) reported statistically significant differences between the two lumbar approaches at L4, with the transverse approach showing better results in canine cadavers [4]. One possible explanation for the lack of differences in injectate spread observed in the present study could be related to anatomical differences between species. Our findings are consistent with those reported in canine cadavers by Herrera-Linares et al. (2024), who found no statistically significant differences when comparing both approaches at the thoracolumbar level [25]. Nevertheless, the anatomical target described by Herrera-Linares et al. (2024) was the transverse process of T12; therefore, anatomical differences between the thoracolumbar and lumbar regions make direct comparisons difficult. It should also be noted that Cavalcanti et al. (2022), in a cadaveric study in dogs comparing longitudinal and transverse approaches at the level of T12, concluded that the transverse approach was superior when the mammillary process was used as a landmark [24].

The transverse process of L3 was used as the anatomical landmark for the longitudinal approach in our study to allow a direct comparation with the procedure described by Nobre et al. (2025) in feline cadavers. On the other hand, the mammillary process was used for the transverse approach, obtaining images comparable to those reported by Medina-Serra et al. (2021) and Cavalcanti et al. (2022) in canine cadavers [4,23,24]. A linear transducer was used, allowing accurate identification of the anatomical structures, in agreement with previous studies using both the longitudinal and the transverse approach [2,3,4,26]. In the present study, visualization of both the target structures and the needle was considered adequate for both approaches. All injections were considered successful based on accurate ultrasound-guided needle placement and correct distribution of the injectate at the intended anatomical target. When comparable results are obtained between approaches, potential differences in procedural aspects may be considered. In human studies, the transverse approach has been suggested as an alternative to the longitudinal technique [6,11,39]. In this view, the mamillary process is clearly identified and serves as a depth limit, reducing the risk of inadvertent pleural injury. However, to the authors’ knowledge, current veterinary evidence does not demonstrate a clear difference in safety outcomes. Another important consideration is that, in the transverse approach, the size of the transducer may represent a practical limitation, particularly in small specimens, where the reduced surface area can hinder optimal probe positioning and image acquisition.

During dissection, a median of 2 DBSN was observed stained, which is consistent with the findings of Nobre et al. (2025), who compared two injectate volumes and reported a mean of 2.8 DBSN stained at the lumbar level with 0.4 mL kg^−1^, and 4.5 DBSN with 0.6 mL kg^−1^ [23]. Similar results were also reported by Medina-Serra et al. (2021) in dogs at the lumbar level using a transverse approach [4]. However, in the present study, a comparable number of DBNS were also stained using the longitudinal approach, whereas Medina-Serra et al. (2021) reported a median of 0 (0–3). Herrera-Linares et al. (2024), using a volume of 0.6 mL kg^−1^ at the thoracolumbar level, differentiated between lateral and medial DBSN reporting a slightly higher number of stained nerves, likely due to the greater volume administered compared to our study [25]. Similarly, Cavalcanti et al. (2022), at the T12 level in dogs, also distinguished between lateral and medial DBSN. However, these authors observed a greater staining of the lateral branches with the transverse approach and greater staining of the medial branches with the longitudinal approach [24]. In our cadavers, as it has been previously reported in cadaveric studies in cats [23,26], due to the reduced size of the DBSN in felines, it was impossible to identify the lateral and medial DBSN.

A caudocranial spread of the injectate was predominantly showed in the CT images, consistent with the findings of Medina-Serra et al. (2021) and Carrillo-Flores et al. (2025) [4,26]. However, in three injections performed using the longitudinal approach, contrast medium was observed below the transverse process, corresponding to the same cases in which partial staining of the VBSN of L3 was identified. This fact confirms our secondary hypothesis. The ventral distribution of the injectate below the middle layer of the thoracolumbar fascia has been previously described in cats [23,26], dogs [25] and horses [20] in the thoracic, thoracolumbar and lumbar regions. One hypothesis explaining this ventral spread is that the regions through which the DBSN and vessels course within the thoracolumbar fascia may present areas of lower resistance and increased permeability, thereby facilitating the spread of injectate to adjacent compartments. However, the potential diffusion of the injectate through the connective tissue should also be considered [40]. An important fact to consider is that this finding was only observed with the longitudinal approach, suggesting that an additional contributing factor could be accidental puncture of the thoracolumbar fascia upon contact with the transverse process, thereby promoting ventral distribution of the injectate.

In line with the observations of Portela et al. (2020) and Ferreira et al. (2019), our study did not reveal any staining or evidence of contrast distribution within the sympathetic trunk, the epidural space, or along lymphatic pathways [2,3]. These results suggest a limited spread of the substance in these regions under the conditions evaluated. However, contrasting evidence has been reported in other investigations, which have documented the presence of staining in the sympathetic trunk [26], epidural spread [4,25], and lymphatic migration [19,25]. These discrepancies may reflect differences in methodology, injection volume, or anatomical variability, highlighting the need for further research to clarify the factors influencing distribution patterns.

The volume used in the present study (0.4 mL kg^−1^), has been previously employed in cats [23,26,41,42,43], and is equivalent to a dose of 2 mg kg^−1^ of bupivacaine or ropivacaine 0.5% [4,44,45]. The administration of larger volumes could potentially surpass the recommended doses for these two commonly used local anesthetics in cats [45,46]. Fascial plane blocks are intrinsically volume-dependent techniques, where the use of greater volumes is often necessary to achieve adequate spread. One frequently employed strategy to accommodate higher volumes without exceeding dose limits is the dilution of local anesthetics. Nevertheless, it should be emphasized that increased dilution generally results in a shorter duration of effect and reduced analgesic potency. Another factor to consider is the density of the injectate mixture containing MB, lidocaine, and iopromide compared to a standard local anesthetic solution. Injectate density has been shown to influence distribution and diffusion through the connective tissue layers of a fascia [40]. In this study, lidocaine was incorporated to approximate the density of conventional local anesthetics. Despite this, it is important to recognize that the presence of both iopromide and MB alters the overall density of the mixture, thereby affecting its spread and tissue diffusion. It has been recently reported that the diffusion capacity of local anesthetics is greater than that of MB and iodinated contrasts [47]. This observation should be considered, as it may help explain why the analgesic effects of regional blocks observed in clinical studies often surpass those predicted by cadaveric experiments.

Several limitations of this study should be acknowledged. First, the small sample size may have influenced the results and limits the generalizability of the findings. No formal sample size calculation or statistical power analysis was performed, which may limit the ability to detect true differences. Furthermore, no correction for multiple comparisons was applied. Consequently, the isolated statistically significant finding observed for L1 DBSN staining should be interpreted with caution, given the number of comparisons performed and the limited sample size. The use of cadaveric specimens introduces additional constraints, as the absence of physiological factors such as blood perfusion, lymphatic drainage, muscle tone, and ventilation could have affected the spread of the dye and contrast medium. Furthermore, the freezing and thawing process may alter tissue properties, potentially modifying injectate distribution. In addition, bilateral injections within the same cadaver may have allowed cross-distribution of the injectate between sides, potentially influencing the observed spread. Although minimal, the intramuscular distribution of the injectate may have slightly reduced its overall spread. This finding is likely related to the small dimensions of feline fascial planes compared with the size of the needle bevel, making complete confinement of the injectate within the interfascial plane challenging. Lidocaine was incorporated to approximate the density of clinically used solutions. However, the addition of MB and iopromide increases the overall density, which may have further influenced the diffusion and spread. Anatomical dissections themselves, involving disruption of tissue barriers, could have inadvertently facilitated distribution beyond what would occur in vivo. However, the confirmation of contrast distribution by CT diminishes this possibility. Finally, the dissections were conducted by non-blinded personnel, which introduces the possibility of observational bias in the interpretation of the findings. Collectively, these limitations should be considered when extrapolating the results to clinical scenarios and highlight the need for cautious interpretation of cadaveric studies.

## 5. Conclusions

Ultrasound-guided lumbar ESP blocks, whether performed using a longitudinal or transverse approach, are feasible techniques in feline cadavers. Neither approach proved superior to the other, as both consistently allowed injectate spread through the epaxial musculature, reaching the DBSN. These findings are limited to cadaveric conditions and should be interpreted as evidence of anatomical feasibility rather than clinical efficacy. Further studies in live animals are needed to determine the actual analgesic effectiveness, optimal dosing, and safety of these techniques.

## Figures and Tables

**Table 2:** Comparison of results between both ESP block approaches.

Variable	Longitudinal Approach	Transverse Approach
Vertebral bodies	4 (3–5)	4 (3–5)
Ventral migration of MB	25%	0%
Epidural space	0	0
Epaxial musculature staining	100%	100%
DBSN staining	2 (1–2)	2 (1–3)
VBSN staining	0 (0–2)	0 (0–0)
Sympathetic trunk staining	0	0

**Table 3:** Comparison of contrast medium distribution between approaches.

Vertebral Body	Longitudinal Approach	Transverse Approach	*p*-Value
T13	0%	8.33%	0.317
L1	33.33%	66.66%	0.110
L2	100%	91.66%	0.317
L3	100%	100%	1.000
L4	100%	100%	1.000
L5	50%	50%	1.000

**Table 4:** Comparison of methylene blue distribution in dorsal branches of spinal nerves. DBSN: dorsal branches of the spinal nerves. * Statistically significant differences between approaches.

DBSN	Longitudinal Approach	Transverse Approach	*p*-Value
L1	0%	25%	0.033 *
Partial L1	0%	8.3%	—
L2	66.66%	75%	0.660
L3	91.66%	91.66%	0.514
Partial L3	8.33%	8.33%	—
L4	8.33%	25%	0.540
Partial L4	8.33%	0%	—

## Data Availability

The original contributions presented in this study are included in the article. Further inquiries can be directed to the corresponding author.

## Abbreviations

The following abbreviations are used in this manuscript:

ESP	Erector Spinae Plane
DBSN	Dorsal Branches
VBSN	Ventral Branches
L3	Third Lumbar Vertebra
L7	Seventh Lumbar Vertebra
TP	Transverse Processes
MB	Methylene Blue
CT	Computed Tomography
Cr	Cranial
Cd	Caudal
D	Dorsal
V	Ventral
Lt	Lateral
M	Medial
L	Left
R	Right
Mam	Mamillary Process
Mm	Multifidus Muscle
